# Editorial: Modern out-of-hospital emergency medical services: organization, function, challenges, and future directions

**DOI:** 10.3389/fpubh.2026.1940095

**Published:** 2026-09-04

**Authors:** Krzysztof Marek Mitura, Piotr Konrad Leszczyński, Sławomir Dariusz Szajda

**Affiliations:** 1 Independent Public Health Care Center RM-MEDITRANS Emergency Station and Sanitary Transport in Siedlce, Siedlce, Poland; 2 Faculty of Medical and Health Sciences, University in Siedlce, Siedlce, Poland; 3 Department of Emergency Medicine, Collegium Medicum, University of Warmia and Mazury in Olsztyn, Olsztyn, Poland

**Keywords:** emergency medical service, emergency medical team, EMS, EMT, OHEMS, out-of-hospital emergency medical service, paramedic, prehospital

The primary aim of establishing Emergency Medical Services (EMS) is to save lives and protect people's health in emergencies. The World Health Organization (WHO) indicates that EMS involves the appropriate allocation of equipment and personnel to enable the effective and coordinated provision of medical assistance in emergency situations (1). WHO distinguishes between two main components of EMS: out-of-hospital emergency medical services (OHEMS) and in-hospital emergency medical services. The out-of-hospital phase of EMS begins when an emergency call is received and ends when the patient is handed over to hospital personnel.

This Research Topic is devoted to analyzing the organization and functioning of modern OHEMS, identifying the challenges they face, and outlining directions for their future development. It also provides an overview of current epidemiological trends associated with OHEMS interventions, emergency procedures, and innovative technological solutions implemented in practice. In line with these objectives, the Research Topic brings together interdisciplinary studies that contribute to a deeper understanding of the optimization of OHEMS.

The articles published as part of this Research Topic address various aspects of OHEMS. The research presented focuses on three main areas: the organization of OHEMS, the analysis of OHEMS interventions, and modern solutions that can be applied within OHEMS.

The organization and structure of modern OHEMS vary from country to country and even from region to region (2, 3). At present, these systems are based primarily on two fundamental organizational models: the Anglo-American and Franco-German models (3, 4). In practice, these models often overlap, and many countries have developed their own solutions. The organization of OHEMS has a significant impact on the effectiveness of care, and differences in system structure, resources, procedures, staff training, and coordination of activities directly affect patient outcomes (5, 6). Castro-Delgado et al. highlight the potential of integrating EMS with primary health care in rural settings; Mitura et al. demonstrate both the strengths and remaining organizational limitations of a nationally standardized OHEMS model; Zahorka et al. show how modeling and decision-support methods could improve resource allocation while emphasizing the persistent gap between methodological innovation and practical implementation; Żuratyński and Haratake illustrate how automated external defibrillator (AED) registries and legal frameworks can function as coordinated system-level interventions; and, finally, Zang et al. remind us that technological and organizational innovation ultimately depends on appropriately trained, supported, and resilient professionals. Collectively, these findings suggest a transition from a predominantly transport- and response-time-oriented paradigm toward integrated, data-driven, patient-centered, and outcome-oriented OHEMS. However, substantial knowledge gaps remain, particularly regarding the prospective evaluation of system-level interventions, multidimensional quality indicators, equity, workforce sustainability, and the translation of advanced modeling and digital technologies into routine clinical practice.

Two published articles focused on the World Health Organization Emergency Medical Teams (WHO EMTs). WHO EMTs are teams of healthcare professionals, including physicians, nurses, and paramedics, who provide rapid, high-quality medical care to populations affected by disasters, epidemics, and other emergency situations. These teams, classified and certified by the WHO, provide standardized, self-sufficient, and coordinated support to healthcare systems. Zwanenburg et al. show that governmental EMTs operating under common WHO standards can adopt substantially different organizational, workforce, activation, logistical, and financing models, whereas Abdelaziz et al. demonstrate that internationally defined standards may be difficult to implement when they are insufficiently adapted to resource-constrained and unstable settings. Their findings highlight the importance of strengthening leadership, logistics, and human resources, as well as addressing barriers to classification and implementation. Together, these articles suggest that effective development of WHO EMTs requires a balance between adherence to internationally defined standards and flexibility to accommodate national and regional contexts, supported by adequate leadership, resources, and organizational capacity.

Another group of publications comprises articles analyzing interventions performed by EMS teams. Such analyses are important both for the quality of patient care and for the functioning of OHEMS as a whole. They make it possible not only to assess the quality of services provided but also to identify areas requiring improvement and implement measures to enhance the effectiveness of EMS (7). Contemporary OHEMS must increasingly respond to a highly heterogeneous patient population while maintaining equitable access to advanced, evidence-based care. The findings of Czyżewski et al., Leszczyński et al., and Mitura et al. converge in demonstrating that prehospital management is shaped not only by patients' acute clinical conditions but also by age, sex, geographic location, and the organizational model through which care is delivered. Helicopter Emergency Medical Service (HEMS) may provide an important mechanism for overcoming geographic inequalities and delivering advanced interventions, particularly in rural areas and among patients with time-critical conditions, while differences between ground and air teams indicate that access to specific interventions may still depend on service configuration. At the same time, observed differences in management according to age and sex highlight the need for more individualized approaches to prehospital assessment and decision-making. Importantly, these articles highlight the need for a more comprehensive understanding of EMS care, incorporating frailty, functional status, disease severity, clinical decision-making, and subsequent hospital outcomes.

The final group of articles published in our Research Topic comprises papers on the potential applications of modern technologies in EMS practice. Although properly trained EMS personnel are competent in diagnosing and treating a wide range of illnesses and medical emergencies, EMS teams operating at the scene of an incident often face limited diagnostic capabilities and patients presenting with non-specific symptoms (8, 9). Consequently, the use of modern tools by EMS teams, such as telemedicine, artificial intelligence, and digital clinical decision-support systems, is becoming increasingly important. These technologies can significantly improve the effectiveness of EMS operations by enhancing decision-making, reducing response times, and enabling more accurate assessment of patients' conditions (10, 11). At the same time, it should be emphasized that technologies incorporating artificial intelligence (AI), despite their promising results, are not yet fully ready for routine clinical use (12). Huo et al. demonstrate how 5G-enabled connectivity can reduce information and coordination delays in time-critical cardiovascular and cerebrovascular emergencies, while Song et al. show that focused assessment with sonography for trauma (FAST) portable point-of-care ultrasonography can extend diagnostic capabilities to resource-limited environments. Zhang et al. further illustrate how unmanned aerial vehicles (UAVs) may overcome spatial limitations by rapidly delivering AEDs, medications, blood products, and other emergency resources. Taken together, these articles demonstrate that technological innovation in OHEMS is increasingly addressing three interconnected dimensions of prehospital care: obtaining clinically relevant information at the scene, transmitting that information rapidly across organizational boundaries, and delivering essential medical resources to patients despite geographic and infrastructural barriers.

OHEMS is a key component of healthcare and public health systems. Consequently, to adapt to societal expectations and contemporary challenges, OHEMS should undergo continuous organizational development, respond to evolving epidemiological trends and emerging threats, and make effective use of modern technologies.

As demonstrated by the articles included in this Research Topic, a resilient OHEMS should be understood as an integrated system capable of adapting to changing circumstances. Organizational integration, a trained and supported workforce, effective coordination, community-based resources, data-driven planning, and appropriately evaluated technologies can provide mutually reinforcing layers of preparedness. By focusing on these issues, the published papers may contribute to the development of best practices, strengthen the resilience of emergency care systems, and ultimately improve population health outcomes during crisis situations. At the same time, future research should move toward whole-system measures of resilience, including continuity of services, equity, workforce capacity, patient outcomes, cost-effectiveness, and the ability to maintain essential care when normal operating conditions are disrupted.

## References

[1] World Health Organization. Regional Office for Europe & European Union. Emergency Medical Services Systems in the European Union: Report of an Assessment Project Co-ordinated by the World Health Organization. Copenhagen: WHO Regional Office for Europe (2008). Available online at: https://iris.who.int/items/94329368-b104-4a7c-8507-11b15934d358 (Accessed June 10, 2026).

[2] Thygerson S Memmott G Chaney R. A global emergency: identifying priorities for reforming international emergency medical systems. Med Res Arch. (2023) 11:1–11. doi: 10.18103/mra.v11i5.3922

[3] Lansiaux E Cozzi N Wacht O Travers S Drouin E Wiel E. Scoop and treat: from an historical controversy to the emergency future. Front Disaster Emerg Med. (2024) 2:1340348. doi: 10.3389/femer.2024.1340348

[4] Al-Shaqsi S. Models of international emergency medical service (EMS) systems. Oman Med J. (2010) 25:320–3. doi: 10.5001/omj.2010.92 22043368 PMC3191661

[5] Bukhari EM Jurays NS Alarmati ST Almalki SN Alsobehi NA Turjoman L . Prehospital interventions, early detection, and their impact on survival outcomes in patients with sepsis: a systematic review. Prehosp Emerg Care. (2026) 30:795–803. doi: 10.1080/10903127.2025.2594601 41334884

[6] Al Abbas HMA Al Hussin YMM Al Hussain AM Alabbas MAS Al-Duways RM Alhareth HSM . Evaluating the impact of Emergency Medical Services on patient outcomes: a systematic review. J Ecohumanism. (2024) 3:9048–54. doi: 10.62754/joe.v3i8.5522

[7] Haugland H Uleberg O Klepstad P Krüger A Rehn M. Quality measurement in physician-staffed emergency medical services: a systematic literature review. Int J Qual Health Care. (2019) 31:2–10. doi: 10.1093/intqhc/mzy106 PMC6387994 29767795

[8] Leszczyński P Muraczyńska B Wejnarski A Baczewska B Malm M Drop B. Improving the quality of training paramedics by means of cadavers - a pilot study. BMC Med Educ. (2021) 21:67. doi: 10.1186/s12909-021-02498-x PMC7836173 33494736

[9] Mitura KM Snarska J Celinski D Maslach D Leszczynski PK Binkowska A . ICD-10 classification in the practice of emergency medical teams: new insights. Emerg Med Int. (2024) 2024:8506561. doi: 10.1155/2024/8506561 38784856 PMC11115992

[10] Kim Y Groombridge C Romero L Clare S Fitzgerald MC. Decision support capabilities of telemedicine in emergency prehospital care: systematic review. J Med Internet Res. (2020) 22:e18959. doi: 10.2196/18959 33289672 PMC7755537

[11] Cimino J Braun C. Clinical research in prehospital care: current and future challenges. Clin Pract. (2023) 13:1266–85. doi: 10.3390/clinpract13050114 37887090 PMC10605888

[12] Semeraro F Schnaubelt S Olasveengen TM Bignami EG Böttiger BW Fijačko N . European Resuscitation Council Guidelines 2025 system saving lives. Resuscitation. (2025) 215(Suppl 1):110821. doi: 10.1016/j.resuscitation.2025.110821 41117570

